# Clinicopathological features and outcome in advanced colorectal cancer patients with synchronous *vs* metachronous metastases

**DOI:** 10.1038/sj.bjc.6605737

**Published:** 2010-06-15

**Authors:** L J M Mekenkamp, M Koopman, S Teerenstra, J H J M van Krieken, L Mol, I D Nagtegaal, C J A Punt

**Affiliations:** 1 Department of Medical Oncology, Radboud University Nijmegen Medical Centre, P.O. Box 9101, Nijmegen 6500 HB, The Netherlands; 2 Department of Pathology, Radboud University Nijmegen Medical Centre, P.O. Box 9101, Nijmegen 6500 HB, The Netherlands; 3 Department of Medical Oncology, University Medical Center Utrecht, Utrecht, The Netherlands; 4 Department of Epidemiology, Biostatistics and Health Technology Assessment, Radboud University Nijmegen Medical Centre, P.O. Box 9101, Nijmegen 6500 HB, The Netherlands; 5 Trial Office Comprehensive Cancer Centre East (IKO), Nijmegen, The Netherlands

**Keywords:** synchronous metastases, metachronous metastases, colorectal cancer, prognostic factors, chemotherapy

## Abstract

**Background:**

Synchronous metastases of colorectal cancer (CRC) are considered to be of worse prognostic value compared with metachronous metastases, but only few and conflicting data have been reported on this issue.

**Methods:**

We retrospectively investigated patient demographics, primary tumour characteristics and overall survival (OS) in 550 advanced CRC patients with metachronous *vs* synchronous metastases, who participated in the phase III CAIRO study. For this purpose only patients with a prior resection of the primary tumour were considered.

**Results:**

The clinical and pathological characteristics associated with poor prognosis that we observed more often in patients with synchronous metastases (*n*=280) concerned an abnormal serum lactate dehydrogenase (LDH) concentration (*P*=0.01), a worse WHO performance status (*P*=0.02), primary tumour localisation in the colon (*P*=0.002) and a higher T stage (*P*=0.0006). No significant difference in median OS was observed between patients with synchronous metastases and metachronous metastases (17.6 *vs* 18.5 months, respectively, *P*=0.24).

**Conclusion:**

Despite unfavourable clinicopathological features in patients with synchronous metastases with a resected primary tumour compared to patients with metachronous metastases, no difference in the median OS was observed. Possible explanations include a (partial) chemoresistance in patients with metachronous disease because of previous adjuvant treatment, whereas differences between the two groups in screening procedures resulting in a lead time bias to diagnosis or in prognostic molecular markers remain speculative.

Approximately 20% of colorectal cancer (CRC) patients present with synchronous distant metastases at the initial diagnosis, and about 50% of the patients without metastases at presentation develop distant metastases within 3 years of diagnosis (McArdle, 2000). For patients with unresectable metastatic CRC there are no curative options, but a significant benefit in median overall survival can be achieved with palliative systemic treatment (Golfinopoulos *et al*, 2007). This treatment consists of cytotoxic chemotherapy (fluoropyrimidines, oxaliplatin, irinotecan) and targeted therapy (VEGF and EGFR antibodies).

Only few data have been reported on the prognostic role of synchronous and metachronous metastases in patients with advanced CRC treated with chemotherapy, and the results are conflicting. Moreover, there is no consensus about the definition of synchronous and metachronous disease. Synchronous metastases were defined as metastases detected by pre-operative screening or during resection of the primary tumour (Manfredi *et al*, 2006; Miller *et al*, 2007; van der Pool *et al*, 2009), and occurring within 3 (Ng *et al*, 2009), 6 (Wang *et al*, 2007; Pantaleo *et al*, 2008) or 12 months (Tsai *et al*, 2007; Bockhorn *et al*, 2008) of the initial diagnosis of CRC. It is not clear whether patients with synchronous *vs* metachronous metastases may represent two different categories of CRC. Only in some surgical intervention trials the clinicopathological features have been compared between patients with metachronous and synchronous metastases (Tsai *et al*, 2007; Wang *et al*, 2007; Ng *et al*, 2009). However, these studies involved small numbers of patients, and only limited clinical and pathological features were evaluated.

In a review of 143 phase II and III studies with 21 214 metastatic colorectal cancer patients, metachronous *vs* synchronous metastases were reported as baseline characteristics in only 18 studies (Sorbye *et al*, 2007). Consequently, few data are available on the prognostic value of this parameter with conflicting results (Nordic Gastrointestinal Tumor Adjuvant Therapy Group, 1992; Graf *et al*, 1994; Freyer *et al*, 2000; Saltz *et al*, 2000; Tournigand *et al*, 2004; Colucci *et al*, 2005).

To our knowledge, this is the first large retrospective analysis on the clinical and pathological characteristics of advanced CRC patients with metachronous *vs* synchronous metastases, and their correlation with outcome. Data were obtained from the phase III CAIRO study of the Dutch Colorectal Cancer Group (DCCG) (Koopman *et al*, 2007a).

## Materials and methods

### Patients

Data were used from the phase III CAIRO study of the DCCG (Koopman *et al*, 2006, 2007a). In this study patients were randomised between sequential and combination treatment with capecitabine, irinotecan and oxaliplatin. Stratification parameters included WHO performance status, serum lactate dehydrogenase (LDH), prior adjuvant therapy, predominant localisation of metastases and participation institution. Assessment of tumour response was scheduled every three cycles (9 weeks) according to RECIST criteria (Therasse *et al*, 2000). Follow-up after completion of treatment was performed every 3 months until death. The primary endpoint was overall survival.

Patients were divided into synchronous and metachronous disease, with synchronous disease defined as distant metastases occurring within, and metachronous disease beyond 6 months of the primary diagnosis of CRC. For two reasons only patients in whom a resection of the primary tumour had been performed were included in the analysis. First, tissue of the primary tumour was required for histopathological review. Second, the arguments for non-resection may greatly vary from patients with an asymptomatic primary and excellent performance status to patients with a symptomatic primary with extensive metastases and poor performance status in whom a delay in systemic treatment is not warranted. These arguments are often not recorded in the patients’ files.

### Pathological procedures and parameters

Standardised pathology examination was performed in the pathology laboratories of the referring hospitals. The maximum diameter was noted with an invasion depth described in terms of the T classification and lymph node stage in terms of the N classification (Sobin and Fleming, 1997). All reports of these examinations with haematoxylin and eosin-stained coupes of the primary tumours were collected. Histopathological review was carried out by two independent observers. If the scoring was not unambiguous, the two observers discussed until agreement was reached. Classification of the tumours was performed using the World Health Organization guidelines (Hamilton and Aaltonen, 2000). A tumour was considered to be of the mucinous type when at least 50% of the tumour was mucinous. The tumours were graded according to the grade of differentiation into well, moderate and poor adenocarcinomas on the basis of the part of poorest differentiation in the tumour. Growth pattern, the presence of inflammatory reaction and fibroblastic reaction were assessed according to Jass *et al* (1987). The mismatch repair system status was determined by immunohistochemistry and microsatellite instability (MSI) analysis (Koopman *et al*, 2009).

### Statistical analysis

The comparison of patient and primary tumour characteristics between patients with synchronous and metachronous metastases was done using Wilcoxon's rank sum test or *χ*^2^-test where appropriate. The progression-free survival (PFS) for first line treatment was calculated from the date of randomisation to the first observation of disease progression or death from any cause. Overall survival and PFS curves were estimated using the Kaplan–Meier method and compared with the log-rank test. Multivariate analysis of survival was performed by means of a Cox proportional hazard model. Patients were considered evaluable for response if they had completed at least three cycles of chemotherapy. Overall response was defined as partial response or complete response. Disease control was defined by stable disease with a duration of more than 4 months or partial response or complete response (Therasse *et al*, 2000). Differences in response and disease control rates were analysed by a *χ*^2^ (univariate) model. All tests were two-sided and *P*-values of less than 0.05 were considered statistically significant. The analyses were performed using SAS 8.2 software.

## Results

### Patient characteristics

In 550 of 803 eligible patients in the CAIRO study, a resection of the primary tumour was performed and material for histological review of the primary tumour was available, and these 550 patients were included in this analysis. Compared to the metachronous group (*n*=270), patients with synchronous metastases (*n*=280) were younger (*P*<0.0001), had more often an abnormal serum LDH at randomisation (*P*=0.01) and more often the liver as predominant site of metastases (*P*<0.0001). Primary tumour localisation in the colon (*P*=0.002), a worse WHO performance status at randomisation (*P*=0.02) and no previous adjuvant chemotherapy (*P*<0.0001) were more frequently observed in patients with synchronous metastases (Table 1).

### Primary tumour characteristics

Tumours of patients with synchronous metastases had larger diameters (*P*=0.007), a higher T (*P*=0.0006) and N stage (*P*<0.0001), absent or little lymphoid reaction (*P*=0.04) and more frequently a diffuse infiltration pattern (*P*=0.02) than patients with metachronous disease. There were no significant differences between the synchronous and metachronous group in terms of classification, differentiation grade, MSI status, and fibroblastic reaction surrounding the tumour (Table 2).

### Correlation of clinical and pathological characteristics with outcome

The effect of clinical and pathological characteristics on median overall survival (OS) was evaluated. In the overall population of 550 patients, the following parameters significantly correlated with the median OS: predominant liver localisation of metastases yes *vs* no (17.9 *vs* 19.5 months, respectively; *P*=0.02), WHO performance status 2 *vs* 0–1 (6.2 *vs* 18.5 months, respectively; *P*<0.0001), serum LDH concentration abnormal *vs* normal (12.8 *vs* 21.3 months, respectively; *P*<0.0001) and number of metastatic sites involved >2 *vs* 2 *vs* 1 (12.4 *vs* 18.0 *vs* 21.4 months, respectively; *P*<0.0001). In the effect on median OS a significant trend was observed for the following pathological characteristics of the primary tumour: T4 *vs* T3 *vs* T1-2 (14.3 *vs* 18.9 *vs* 21.9 months, respectively; *P*=0.03), N2 *vs* N1 *vs* N0 (14.4 *vs* 18.9 *vs* 20.7 months, respectively; *P*=0.003), mucinous carcinoma *vs* adenocarcinoma with mucinous component *vs* adenocarcinoma (13.5 *vs* 13.7 *vs* 19.3 months, respectively; *P*=0.006) and differentiation grade poor *vs* moderate *vs* well (14.8 *vs* 20.4 *vs* 24.9 months, respectively; *P*=0.0001). By univariate analysis no effect on median OS was found for age, gender, site of the primary tumour, prior adjuvant therapy, treatment arm, infiltration pattern, fibroblastic reaction, lymphoid reaction, and MSI status.

In a multivariate model all patient and primary tumour characteristics were included. Independent predictors for median OS in advanced CRC patients, were T stage (*P*=0.04), differentiation grade (*P*=0.01), classification (*P*=0.007), serum LDH at randomisation (*P*<0.0001), WHO performance status (*P*=0.01), site of the primary tumour (*P*=0.0008), gender (*P*=0.03) and metastatic sites involved (*P*<0.0001) (Table 3).

### Outcome in patients with metachronous *vs* synchronous metastases

**Figure 1:**
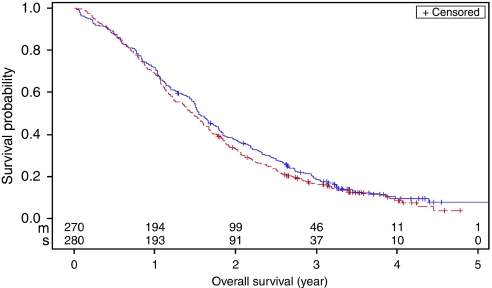
Kaplan–Meier curve for overall survival of advanced CRC patients with metachronous (---) and synchronous (---) metastases in whom a resection of the primary tumour was performed.

No significant difference in median OS was observed for patients with metachronous *vs* synchronous metastases in univariate analysis (18.5 *vs* 17.6 months, respectively; *P*=0.24) (Figure 1). In addition, to assess a possible effect of tumour burden, we compared the largest diameter of liver metastases between the two groups, and no difference was observed (*P*>0.05, data not shown). In a multivariate model, in which all patient and primary tumour characteristics were included, the hazard ratio for metachronous *vs* synchronous metastases was 1.05 (95% CI 0.81–1.36; *P*=0.74) (Table 3).

The median PFS in first line treatment was not significantly different between patients with metachronous *vs* synchronous metastases (7.2 *vs* 6.6 months, respectively; *P*=0.23). In all, 494 patients were assessable for response in first line treatment: 235 in the metachronous group and 259 in the synchronous group. The overall response rate (complete plus partial tumour response) in first line treatment was significantly better in patients with synchronous metastases compared to patients with metachronous metastases (38 *vs* 28%, respectively; *P*=0.02). The disease control rate (complete plus partial tumour response plus stable disease) in first line treatment was not significantly different between patients with synchronous and metachronous metastases (81 *vs* 87%, respectively; *P*=0.11).

### Interaction of worse prognostic factors in patients with synchronous *vs* metachronous metastases

Patients with synchronous *vs* metachronous metastases in whom a resection of the primary tumour was performed showed significantly different clinical and pathological characteristics. Most of these clinicopathological features were correlated with outcome in the total study population. However, despite the presence of factors associated with poor prognosis, patients with synchronous metastases had no worse survival compared to patients with metachronous metastases.

To find a possible explanation for this observation we analysed whether the median OS of patients with individual clinical and pathological characteristics was significantly different between the synchronous and metachronous group. However, this proved not to be the case (*P*>0.05 for all analyses).

Next, we compared the number of worse prognostic factors *per patient* between the synchronous and metachronous group to detect whether there was a skewed distribution. Again, this analysis showed no significant difference in the distribution of these characteristics per patient between the synchronous and metachronous group (*P*>0.05 for all analyses).

## Discussion

In this retrospective analysis of the phase III CAIRO trial, we observed that CRC patients with synchronous metastases, in whom the primary tumour was resected significantly more often, had clinical and pathological characteristics associated with poor prognosis compared to patients with metachronous metastases.

There is no consensus in the literature on the definition of synchronous *vs* metachronous metastases. We selected a cut-off value of 6 months after the initial diagnosis for two reasons. First, in some patients a staging procedure is performed only after full recovery from surgery of the primary tumour, which may take several months in some patients. A 6-month period will assure adequate classification of these patients. Second, metastases developing during the first 6 months after surgery of the primary tumour probably reflect similar tumour biology compared with metastases detected at initial diagnosis. Therefore, we consider a 6-month cut-off value to be a clinically useful distinction between synchronous and metachronous disease.

The unfavourable clinical characteristics that we observed more often in patients with synchronous disease concerned a worse performance status, an abnormal serum LDH and the colon as the primary site of the tumour. Only the primary site of the tumour has been previously described as being different between synchronous and metachronous disease (Tsai *et al*, 2007; Ng *et al*, 2009). We identified a higher T stage of the primary tumour as an independent worse prognostic factor for median OS, which we observed more in patients with synchronous metastases. This confirms previously reported results of smaller series (Tsai *et al*, 2007; Wang *et al*, 2007; van der Pool *et al*, 2009).

Despite these poor baseline characteristics in patients with synchronous metastases, the median OS was not decreased compared to patients with metachronous metastases. Tsai *et al* (2007) found differences in diameter, number and distribution of liver metastases between patients with synchronous and metachronous disease, and concluded that these characteristics were of significant importance for survival. Tumour burden, as determined by the largest diameter of measurable disease and the number of metastatic sites, were comparable between patients with synchronous and metachronous metastases, indicating that this parameter did not influence our results. However, several other factors may explain this unexpected finding. First, a significant percentage of patients with metachronous metastases were treated with prior adjuvant chemotherapy, whereas patients with synchronous metastases obviously were not. Theoretically, this may have resulted in a (partial) resistance to chemotherapy in the former group. Indeed, we observed a higher overall response rate to first line chemotherapy in patients with synchronous metastases, suggesting that this may compensate the presence of worse prognostic factors in this group. Second, there may be heterogeneity between and also within the groups of patients with synchronous and metachronous disease with regard to symptomatic *vs* asymptomatic disease and, in the latter situation, a lead time bias caused by different time schedules for screening. Third, survival of CRC patients could be influenced by a difference in the presence of prognostic molecular markers between patients with synchronous *vs* metachronous metastases (Pantaleo *et al*, 2008).

Comparing our results with the literature, only few chemotherapy trials performed proportional hazard models to determine the influence of metachronous and synchronous disease on median OS. Several authors showed no prognostic role for these parameters (Kemeny *et al*, 1989; Saltz *et al*, 2000; de Gramont *et al*, 2000; Tournigand *et al*, 2004; Colucci *et al*, 2005), whereas others identified metachronous disease as a favorable prognostic parameter (Nordic Gastrointestinal Tumor Adjuvant Therapy Group, 1992; Graf *et al*, 1994; Freyer *et al*, 2000; Etienne-Grimaldi *et al*, 2008). Our analysis differs from the published literature in one important aspect, in that only patients with a previous resection of the primary tumour were included in the synchronous group. If patients with both resected and nonresected primary tumours were included in the synchronous group, a significant median OS benefit was observed for patients with metachronous *vs* synchronous metastases (Koopman *et al*, 2007b). Therefore, the conflicting results of previous studies on the prognostic role of synchronous disease may be caused by differences among these studies in the status of the resection of the primary tumour. Support for our data is provided by two recent prospective analyses in which no difference in overall survival was observed between patients with resected synchronous *vs* resected metachronous CRC liver and lung metastases, with a resection of the primary tumour having been performed in all patients (Ng *et al*, 2009; van der Pool *et al*, 2009).

In conclusion, despite the presence of factors associated with poor prognosis in patients with synchronous metastases, the parameter of synchronous and metachronous metastases was not of prognostic value in advanced CRC patients in whom a resection of the primary tumour was performed. Possible explanations include a (partial) chemoresistance in patients with metachronous disease because of prior adjuvant treatment, whereas differences between the two groups in screening procedures resulting in a lead time bias to diagnosis or in prognostic molecular markers remain speculative.

## Figures and Tables

**Table 1:** Baseline characteristics

	**Metachronous *n*=270**	**Synchronous *n*=280**	***P*-value**
*Age at randomisation*			
Median (range)	66.0 (31.0–79.0)	62.5 (34.0–81.0)	<0.0001^a^
			
*Gender*			
Male	163 (60%)	179 (64%)	0.43^b^
Female	107 (40%)	101 (36%)	
			
*Predominant localisation metastases*			
Liver	131 (49%)	240 (86%)	<0.0001^b^
Extrahepatic	135 (50%)	39 (14%)	
Unknown	4 (1%)	1 (<1%)	
			
*WHO performance status at randomisation*			
0 and 1	265 (98%)	262 (94%)	0.02^b^
2	5 (2%)	17 (6%)	
Unknown		1 (<1%)	
			
*Serum LDH at randomisation*			
Normal	183 (68%)	161 (58%)	0.01^b^
>ULN	84 (31%)	117 (42%)	
Unknown	3 (1%)	2 (<1%)	
			
*Site of primary tumour*			
Colon	114 (42%)	149 (53%)	0.002^b^
Rectosigmoid	65 (24%)	73 (26%)	
Rectum	91 (34%)	58 (21%)	
			
*Prior adjuvant therapy*			
No	189 (70%)	276 (99%)	<0.0001^b^
Yes	81 (30%)	3 (1%)	
Unknown		1 (<1%)	
			
*Metastatic sites involved*			
1	121 (45%)	141 (50%)	0.63^b^
2	96 (35%)	95 (34%)	
>2	45 (17%)	44 (16%)	
Unknown	8 (3%)		
			
*Treatment arm*			
Sequential	130 (48%)	138 (49%)	0.80^b^
Combination	140 (52%)	142 (51%)	

Abbreviation: ULN=upper limit of normal.

a Wilcoxon rank sum test.

b *χ*^2^.

**Table 2:** Primary tumour characteristics

	**Metachronous *n*=270**	**Synchronous *n*=280**	***P*-value**
*Diameter*			
Median (range)	40.0 (15.0–120.0)	45.0 (15.0–140.0)	0.007^a^
			
*Invasion depth*			
T 1–2	28 (10%)	7 (3%)	0.0006^b^
T 3	187 (69%)	200 (71%)	
T 4	45 (17%)	59 (21%)	
Unknown	10 (4%)	14 (5%)	
			
*Lymph node status*			
N 0	104 (39%)	50 (18%)	<0.0001^b^
N 1	97 (36%)	97 (35%)	
N 2	56 (21%)	113 (40%)	
Unknown	13 (5%)	20 (7%)	
			
*Classification*			
Adenocarcinoma	216 (80%)	217 (78%)	0.53^b^
Adenocarcinoma with mucinous component	28 (10%)	33 (12%)	
Mucinous carcinoma	24 (9%)	24 (8%)	
Other	2 (1%)	6 (2%)	
			
*Differentiation grade*			
Well	11 (4%)	11 (4%)	0.15^b^
Moderate	143 (53%)	125 (45%)	
Poor	115 (43%)	141 (50%)	
Unknown	1 (<1%)	3 (1%)	
			
*Infiltration pattern*			
Circumscribed	69 (26%)	48 (17%)	0.02^b^
Diffuse	199 (74%)	226 (81%)	
Unknown	2 (<1%)	6 (2%)	
			
*Fibroblastic reaction*			
None/little	84 (31%)	76 (27%)	0.39^b^
Extensive	184 (68%)	196 (70%)	
Unknown	2 (1%)	8 (3%)	
			
*Lymphoid reaction*			
None/little	193 (71%)	218 (78%)	0.04^b^
Extensive	74 (27%)	55 (20%)	
Unknown	3 (1%)	7 (2%)	
			
*MSI status*			
pMMR	261 (97%)	271 (97%)	0.94^b^
dMMR	9 (3%)	9 (3%)	

Abbreviations: dMMR=deficient mismatch repair system; pMMR=proficient mismatch repair system.

a Wilcoxon rank sum test.

b *χ*^2^.

**Table 3:** Prognostic value of clinical and pathological characteristics for OS (multivariate analysis)

**Multivariate analysis for OS**	**Hazard ratio (95% CI)**	***P*-value**
*Onset of metastasis*		
Metachronous	1.05 (0.81–1.36)	0.74
Synchronous	R	
		
*Gender*		
Female	0.78 (0.63–0.97)	0.03
Male	R	
		
*Site of primary tumour*		
Colon	1.29 (0.98–1.70)	0.0008
Rectosigmoid	0.78 (0.58–1.04)	
Rectum	R	
		
*WHO performance status at randomisation*		
0 and 1	0.53 (0.32–0.88)	0.01
2	R	
		
*Serum LDH at randomisation*		
>ULN	1.79 (1.44–2.23)	<0.0001
Normal	R	
		
*Number of metastatic sites involved*		
1	0.40 (0.30–0.53)	<0.0001
2	0.55 (0.41–0.75)	
>2	R	
		
*Invasion depth*		
T 1–2	0.69 (0.42–1.12)	0.04
T 3	0.72 (0.55–0.93)	
T4	R	
		
*Classification*		
Adenocarcinoma	1.00 (0.44–2.25)	0.007
Adenocarcinoma with mucinous component	1.56 (0.66–3.68)	
Mucinous carcinoma	1.71 (0.71–4.13)	
Other	R	
		
*Differentiation grade*		
Well	0.69 (0.40–1.20)	0.01
Moderate	0.73 (0.59–0.91)	
Poor	R	

Abbreviations: R=reference group; ULN=upper limit of normal.
